# GP-provided couple-based expanded preconception carrier screening in the Dutch general population: who accepts the test-offer and why?

**DOI:** 10.1038/s41431-019-0516-0

**Published:** 2019-09-30

**Authors:** Juliette Schuurmans, Erwin Birnie, Adelita V. Ranchor, Kristin M. Abbott, Angela Fenwick, Anneke Lucassen, Marjolein Y. Berger, Marian Verkerk, Irene M. van Langen, Mirjam Plantinga

**Affiliations:** 1 Department of Genetics, University of Groningen, University Medical Center Groningen, Groningen, the Netherlands; 2 Faculty of Medicine, Clinical Ethics and Law, University of Southampton, Southampton, UK; 3 Department of Health Psychology, University of Groningen, University Medical Center Groningen, Groningen, the Netherlands; 4 University of Groningen, University Medical Center Groningen, General Practice and Elderly Care, Groningen, the Netherlands; 5 Department of Internal Medicine, University of Groningen, University Medical Center Groningen, Groningen, the Netherlands

**Keywords:** Genetic testing, Ethics, Population screening

## Abstract

Next generation sequencing has enabled fast and relatively inexpensive expanded carrier screening (ECS) that can inform couples’ reproductive decisions before conception and during pregnancy. We previously showed that a couple-based approach to ECS for autosomal recessive (AR) conditions was acceptable and feasible for both health care professionals and the non-pregnant target population in the Netherlands. This paper describes the acceptance of this free test-offer of preconception ECS for 50 severe conditions, the characteristics of test-offer acceptors and decliners, their views on couple-based ECS and reasons for accepting or declining the test-offer. We used a survey that included self-rated health, intention to accept the test-offer, barriers to test-participation and arguments for and against test-participation. Fifteen percent of the expected target population—couples potentially planning a pregnancy—attended pre-test counselling and 90% of these couples proceeded with testing. Test-offer acceptors and decliners differed in their reproductive characteristics (e.g. how soon they wanted to conceive), educational level and stated barriers to test-participation. Sparing a child a life with a severe genetic condition was the most important reason to accept ECS. The most important reason for declining was that the test-result would not affect participants’ reproductive decisions. Our results demonstrate that previously uninformed couples of reproductive age, albeit a selective part, were interested in and chose to have couple-based ECS. Alleviating practical barriers, which prevented some interested couples from participating, is recommended before nationwide implementation.

## Introduction

Next generation sequencing allows fast and relatively inexpensive simultaneous testing for carrier status of many (rare) genetic conditions called expanded carrier screening (ECS) [1]. Deciding what to include in ECS is a complex issue and may depend on for example the target population or the setting in which ECS is offered. As a study by Chokoshvili et al. demonstrates, currently available tests vary greatly in composition of the test-panel [2] and may consist of autosomal recessive (AR), X-linked or in some cases even autosomal dominant conditions. ECS can inform reproductive decisions before and during pregnancy. Couples found to be at increased  risk might wish to consider alternative reproductive options to conceive, e.g. in vitro fertilisation and pre-implantation genetic testing (PGT), non-carrier donor gametes or prenatal testing.

The Genetics Department of the University Medical Centre Groningen (UMCG) in the Netherlands developed and validated a population-based ECS test for a limited set of 50 severe early-onset AR conditions for which no curative treatment is available. Based on the outcome of an international expert meeting, and supported by recent guidelines [3, 4], we developed this gene-panel to evaluate its potential for ECS implementation within the public health system. Whilst in the future, this test could also be complemented with individual carrier screening for X-linked conditions [5, 6], here we chose to focus on AR conditions only, and adopted a couple-based approach. If both members of a couple are carriers for the same AR condition—i.e. carrier-couples—then for each pregnancy there is a risk of one in four or 25% of an affected child. The conditions included in the test carry no known health implications for the individuals in the couple; the only known health implications relate to their future offspring. Previous research among potential users demonstrated an interest in such a test and also identified the general practitioner (GP) as the preferred provider [7, 8]. With these results in mind, we conducted a pilot implementation study in which GPs offered this couple-based ECS to women and their partners from the general population at no financial cost. The main aim of this test-offer was not to encourage as many people as possible to undergo couple-testing. We were primarily interested in how many eligible couples were willing to be informed in more detail about ECS by their GP and how many of such prepared couples made a decision to proceed with testing. This aligns with recommendations from international professional societies which describe the main aim of ECS as to facilitate informed reproductive decision-making [3, 4]. We previously reported that the test-offer is feasible and results in informed choice [5]. Here, we describe the initial interest in this GP-provided couple-based ECS from the target population, the characteristics of couple members who decide to accept and decline the test-offer and their reasons why.

## Methods

### Study design and test-offer

**Fig. 1:**
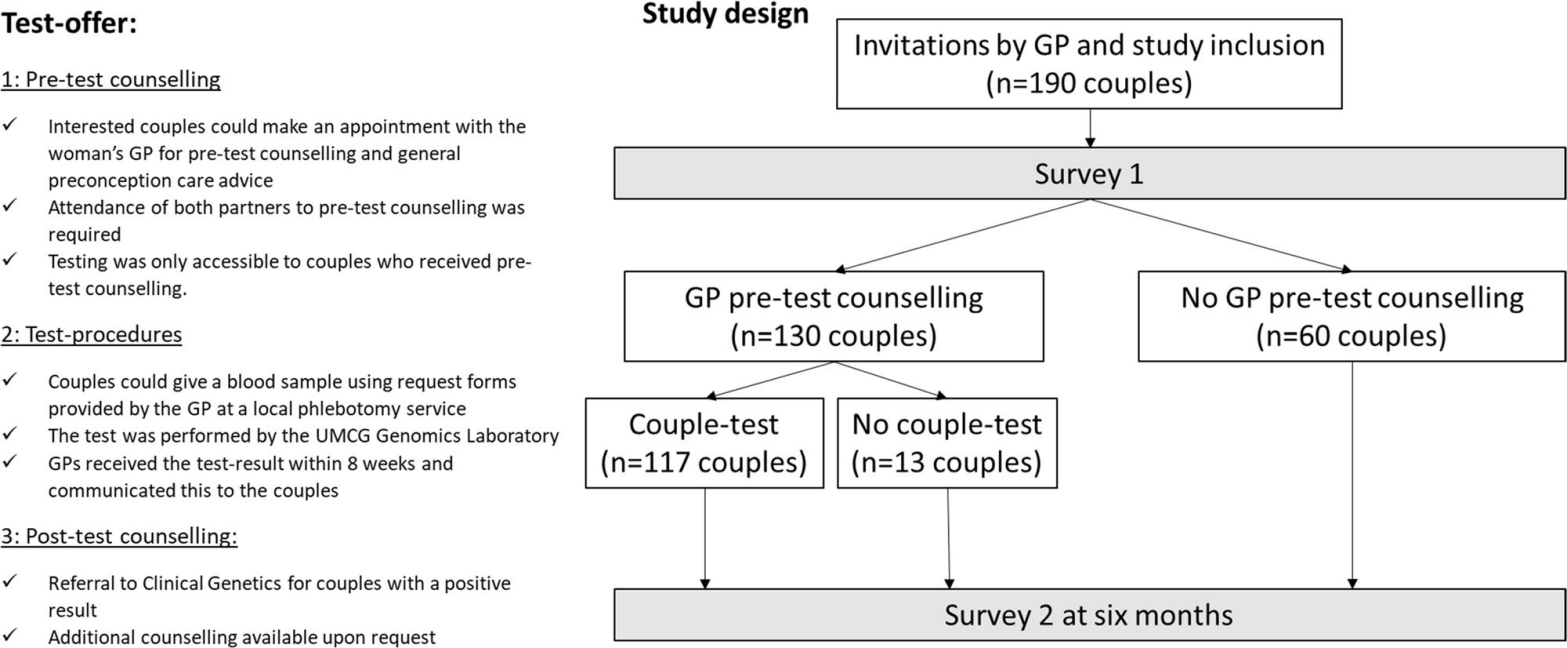
Overview of test-offer and study design

Figure 1 outlines the different elements of our study design and ECS test-offer. Nine GP practices in the catchment area of the UMCG sent out letters to all women aged 18–40 registered with their practices, inviting them to take part in the implementation study. Women were asked to invite their male partners to participate and written consent was requested from both partners. As Fig. 1 shows, all participating couples, regardless of whether they attended pre-test counselling or proceeded with testing, were asked to fill out two online surveys: Survey 1 at study onset and (if they had filled out Survey 1), Survey 2 6 months afterwards. Couples who were interested in the test-offer, could make an appointment for pre-test counselling with the woman’s GP within ~1 month. We asked both partners to attend counselling together, after which they could decide whether they would like to have the screening test.

Prior to the start of the study, all GPs received training to prepare them for the ECS pre-test counselling and the first two counselling sessions for each GP were supervised by a clinical genetics professional. GPs could also refer couples at high a priori risk (e.g. consanguineous couples) and couples needing additional pre-test counselling directly to the Department of Clinical Genetics. Further details about the study design and GP involvement have been reported elsewhere [5]. We also launched a publicly accessible website, www.dragerschapstest.umcg.nl, with general information about the study and the test that also included details on how to ask questions to the research team. For GPs, support from a genetic counsellor from the research team was available throughout the study. The ECS test was offered free of charge to participating couples. If couples received a positive couple result, reproductive options such as PGT and prenatal testing would be covered by statutory health insurance to all the Dutch citizens. The Medical Research Ethics Committee (METc) of the UMC Groningen approved the study protocol (METc 2015/384).

### Recruitment and study inclusion

**Fig. 2:**
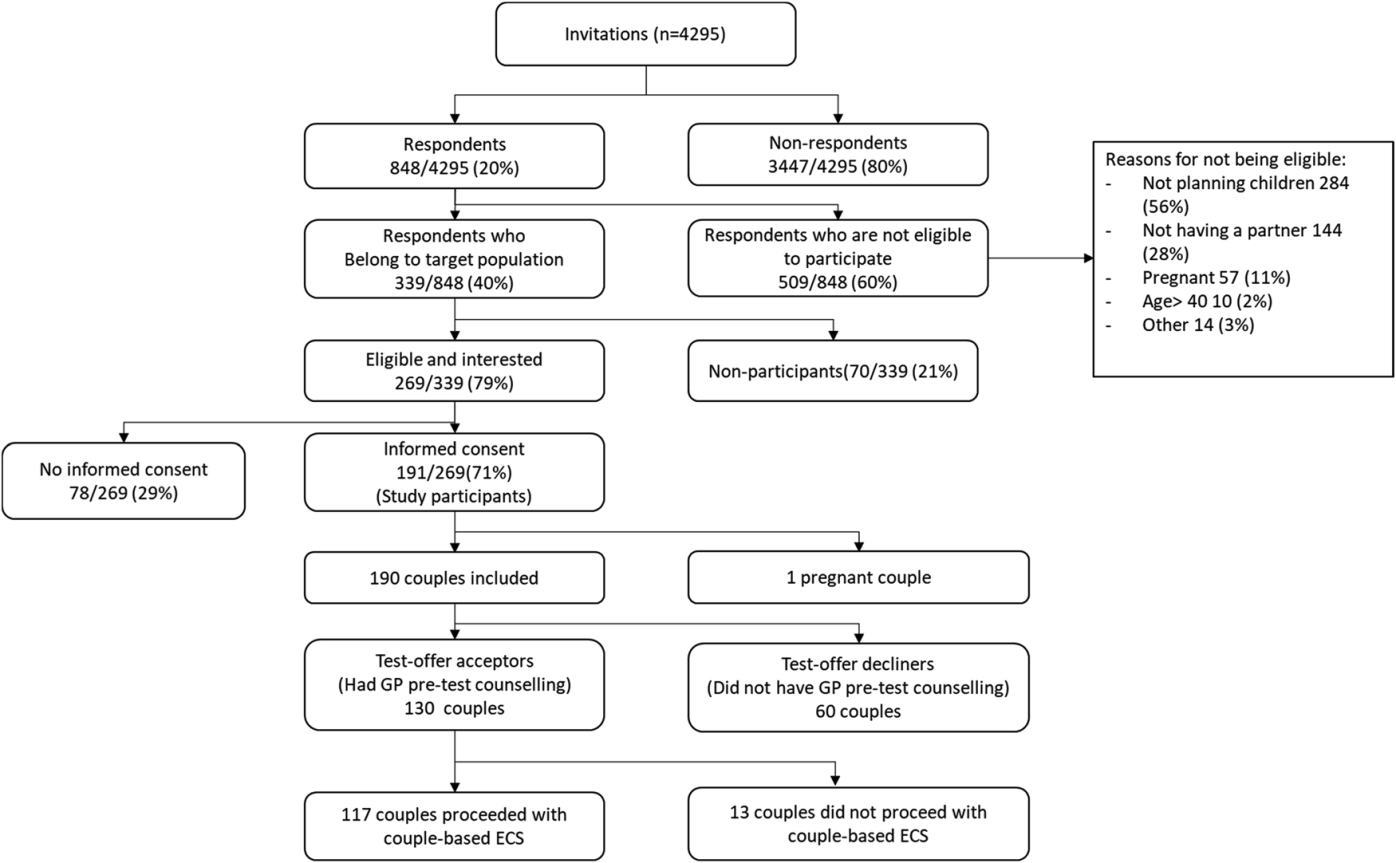
Flow diagram of recruitment and inclusion

Figure 2 displays the recruitment and inclusion of participants in the study. Between January and December 2016, the GPs invited 4295 women aged 18–40 to participate. Women over 40 were excluded from this study because ethical issues may arise due to limited access to PGT for women older than 40 in the Netherlands. All were asked to return the response card to indicate their eligibility and interest in taking part. Women were eligible for participation if they had a male partner, were planning to have children with this partner and were not pregnant. We excluded pregnant women for two main reasons. Firstly, this was the first time GPs were offering ECS and our initial training focused on the least complex pre-test counselling. Secondly, the turn-around-time of the test-result was a maximum of 8 weeks, which limits the time for couples to consider a potential termination of pregnancy in case of a positive test-result. Fourteen women were not eligible for ‘other’ reasons, for example they could not conceive biologically with their partner due to gender affirming treatment. A test-result was considered positive only if both partners have a class IV or V variant in the same recessive disease gene included in the test.

### Materials and measures

Data were collected via questionnaires using the Roqua online tool for confidential clinical data collection [9]. We asked participants (*n* = 190 couples) to fill in the surveys independently from their partners. The survey design was based on research described in [7, 8] that explored attitudes, intentions and reasons for and against accepting couple-based ECS in a hypothetical scenario. The reasons for and against accepting ECS are based on key ethical arguments previously described in [7]. Survey 1 recorded participants’ sociodemographic characteristics, factors related to their relationship and reproduction, their own health and their experiences with (presumed) hereditary conditions, genetic counselling and testing. In addition, we collected data on their intention to participate in testing and their perceived barriers to test-participation. After 6 months, in Survey 2, we collected data on how participants retrospectively viewed their decision about ECS testing and their views on couple-based test-provision.

### Test-offer acceptance and uptake rate

We distinguished test-offer acceptance (defined as attending pre-test counselling by the woman’s GP) from actual ECS test-uptake because the main aim of the test-offer was to inform couples and encourage them to discuss test-participation with the GP if they were interested, an aim separate from the final uptake of the couple-based ECS test itself. We calculated an additional acceptance rate based on the estimated eligible population (couples planning a pregnancy). Previous research showed that ~20% of women between 18 and 40 years of age would be eligible for the test-offer [10, 11]. In our case, we estimated the eligible population as 859 women (or 20% of the 4295 invited women). Thus, in this paper, we use the following three definitions of test-offer acceptance:

1. The proportion of women and their partners who accepted the test-offer as part of the total number of women who were invited (denominator *n* = 4295),
2. the proportion of women and their partners who accepted the test-offer as part of an estimated eligible population (denominator *n* = 859),
3. the proportion of women and their partners who accepted the test-offer (*n* = 130) as part of the total number of women who participated in the study (denominator *n* = 190).

Lastly, the ECS test-uptake rate was calculated as the proportion of couples who proceeded with testing after pre-test counselling.

### Variables included in the survey

#### Characteristics of test-offer acceptors and decliners

##### Sociodemographic characteristics

Age was divided into three categories in a similar way as reported by Plantinga et al. [7]: 18-23-24-33; >33 years of age. Participants’ educational level, marital status and religiosity were classified according to the Statistics Netherlands (CBS) definitions. Educational level was further summarised as: ‘basic’ (finished primary school, lower secondary school or vocational training), ‘intermediate’ (finished higher level secondary school or intermediate vocational training) or ‘high’ (finished higher vocational training or university). Relationship status was classified as ‘marriage or civil partnership’, ‘living together’ or ‘not living together’. Religiosity was measured by asking whether respondents were religious (0 = no, 1 = yes and practising, 2 = yes, but not practising). This was dichotomised into no or yes (including both practising and non-practising).

##### Relationship and reproductive characteristics

Relationship satisfaction was measured on a 10-point scale (1 = very unsatisfied, 10 = very satisfied) [12, 13]. Participants were also asked within what timeframe they were planning to have children and whether they already had children. To be comparable with the other relevant Dutch studies, timing to next pregnancy was adapted from Henneman et al., who dichotomised into <2 years (short term) and >2 years (long term) [14]. We further categorised these into: (<0.5 years, 0.5 years-2 years, 2–5 years, >5 years, unsure)

##### Health status and experiences with hereditary conditions and genetic testing

We asked participants to rate their own health on a 5-point scale (poor, moderate, good, very good and excellent). They were also asked whether they suffered from a chronic condition and were presented with fourteen categories, such as respiratory conditions (e.g. asthma), visual problems and mental health issues (yes/no). In addition to this, we asked respondents to indicate whether they, or any of their family members or friends, suffered from a (presumed) hereditary condition and/or whether they had ever had genetic counselling and testing themselves.

#### Intention, barriers and views on couple-based test-provision

##### Intention (Survey 1)

Intentions towards couple-based ECS before pre-test counselling were measured with the item ‘I intend to accept the offer of this couple-based ECS-test’ on a 7-point scale (unlikely–likely). Intentions were classified into ‘unlikely’ (1-2), ‘neutral’ (3-5) and ‘likely’ (6-7).

##### Barriers (Survey 1)

We collected data about the extent to which participants perceived the time and effort of test-participation, having to make a GP appointment and giving a blood sample, as barriers for taking part in this test-offer. These four items were measured on a Likert scale from 1 to 5 (totally disagree to totally agree).

##### Intention (Survey 2)

We asked test-decliners whether it was a considered decision not to proceed with testing (yes/no). If it was not, they could indicate their reasons why (e.g. we could not come to a common decision as a couple, it just did not happen, I had not thought about it anymore, it was not possible to be at the GP appointment together).

##### Couple-based test-offer (Survey 2)

Participants were also asked to indicate their main preference as to how test-results were disclosed. They were asked to indicate *one* preference out of the following four options: 1: couple results; 2: individual carrier results; 3: no preference; 4: not sure.

#### Arguments for and against accepting the couple-based test-offer

In Survey 1, we asked participants about the reasons why they would accept or decline ECS testing by presenting them with seven arguments in favour and ten arguments against (listed in Tables 3 and 4) taking a couple-based ECS test. We asked all participants which single argument they considered most important in accepting and which single argument they considered most important in declining the test-offer.

### Response cards

In addition, we received explanations of 70 eligible (members of) couples who returned the response card but decided not to take part in the study (and therefore the ECS test).

### Data analysis

Given that partners within a couple might have different views about this couple-based ECS test-offer, individual participants were included in the analysis for all outcome measures apart from acceptance and uptake rate. Only respondents who filled out survey 1 were sent survey 2 and so response rates for survey 2 were calculated based on the number of participants who filled out survey 1. Descriptive data are presented using mean (SD), median (IQR) or numbers (percentages) where appropriate. To compare test acceptors and decliners, unpaired *T*-tests were used for continuous variables and *χ*^2^ tests for categorical variables. Analyses were done using IBM SPSS version 23 (IBM Corp., Armonk, NY, USA).

## Results

### Response and study inclusion

Figure 2 is a flow diagram of the study. We received 848 response cards and in total, 509 women who returned the response card were not eligible (reasons listed in Fig. 2). Seventy eligible women indicated that they did not want to participate. The eligible women who were interested in taking part (*n* = 269) received detailed information about the study, were asked to invite their partner to participate with them and to return consent forms for both of them. After we received their written consent, 191 couples were eventually sent Survey 1. Subsequently, one couple was excluded because they became pregnant before filling out survey no. 1. Thus, in total, we included 190 couples (380 participants), 358/380 (94%) participants returned Survey 1 and 227/358 (63%) participants returned Survey 2.

### Test-offer acceptance and test-uptake rate

In total, 130 couples attended pre-test counselling and 117 of these couples proceeded with testing. This resulted in the following test-offer acceptance and test-uptake rates:

1. Test-offer acceptance was 3% (130/4295) (95% CI 3–4%) of the total invited population (i.e. women aged between 18 and 40 registered with the participating GPs)
2. Test-offer acceptance was 15% (130/859) (95% CI 13-18%) of the estimated eligible population and 68% (130/190) (95% CI 61–75%) for the participants included in this survey study.
3. The uptake rate of the ECS test in participants having attended the GP-consultation was 90% (117/130) (95% CI 84–95%).

### Characteristics of test-offer acceptors and decliners

#### Sociodemographic, relationship and health characteristics

Table 1 displays the characteristics of test-offer acceptors and decliners. The average age in our study sample was 29 (SD 5.5) years and 50% of participants were between 24 and 33 years old. The majority of participants had an intermediate or higher education (93%), and 43% had already finished higher education. Twenty-four percent were religious and 25% were not (yet) living together. The relationship satisfaction rate was a median of 9 out of 10 (IQR 8–9). Fifteen percent already had children (*n* = 55) and sixteen percent were planning a pregnancy within 6 months. Thirty-nine percent reported at least one chronic condition, mainly asthma, migraine or mental health problems, but 97% described their health as good to excellent. Thirty percent had experiences with (supposedly) hereditary conditions in their family or friends, and 13 participants (4%) had previously had genetic counselling and testing. Most of these participants (*n* = 11), made an appointment with the GP to discuss couple-based ECS.

**Table 1:** Sociodemographic characteristics

Sociodemographic characteristics	All *n* = 358	Test-offer acceptors *n* = 259	Test-offer decliners *n* = 99
Age (year) mean (SD)	29.1 (5.5)	29.4 (5.5)	28.7 (5.4)
Gender			
Female	185 (51.7)	130 (49.4)	55 (57.9)
Male	173 (48.3)	129 (49.6)	44 (44.4)
Age category			
18–24	69 (19.3)	46 (17.8)	23 (23.2)
24–32	180 (50.3)	134 (51.7)	46 (46.5)
>33	109 (30.4)	79 (30.5)	30 (30.3)
Religiosity			
Yes	84 (23.5)	65 (25.1)	19 (19.2)
Educational level**			
Basic	25 (7.0)	14 (5.4)	11 (11.1)
Intermediate	178 (49.7)	117 (45.2)	61 (61.6)
High	155 (43.3)	128 (49.4)	27 (27.3)
Marital status			
Married/civil partnership	77 (21.5)	59 (20.8)	18 (18.2)
Living together	196 (54.7)	146 (56.4)	50 (50.5)
Not living together	90 (25.1)	59 (22.8)	31 (31.3)
Children*			
Yes	55 (15.4)	31 (12)	24 (24.2)
Relationship satisfaction*			
Median (IQR)	9 (8–9)	9 (8–9)	8 (8–9)
Timing of next pregnancy*			
<0.5 year	56 (15.6)	35 (13.5)	21 (21.2)
0.5–2 year	103 (28.8)	74 (28.6)	39 (39.4)
2–5 year	126 (35.2)	102 (39.4)	24 (24.2)
≥5 year	36 (10.1)	27 (10.4)	9 (9.1)
Unsure	27 (7.5)	21 (8.1)	6 (6.1)
Self-rated health			
Excellent	90 (25.1)	73 (28.2)	17 (17.2)
Very good	129 (36.0)	95 (36.7)	34 (34.3)
Good	127 (35.5)	83 (32.0)	44 (44.4)
Moderate	12 (3.4)	8 (3.1)	4 (4.0)
Poor	0 (0.0)	0 (0.0)	0 (0.0)
Do you suffer from a chronic condition?			
No	218 (60.9)	162 (62.5)	56 (56.6)
Any experiences with hereditary conditions in your family or friends?			
No experience	252 (70.4)	179 (69.1)	73 (73.7)
Did you have genetic testing and counselling in the past?			
Yes	13 (3.6)	11 (4.2)	2 (2.0)

Test-offer acceptors and decliners were compared using *T*-tests for continuous variables and *χ*^2^ tests for categorical variables. A *p* value of 0.05 was considered statistically significant. *p value <0.05. **p value <0.01

Test-offer acceptors and decliners differed significantly in the highest level of education achieved: test-offer acceptors more frequently had a higher educational level. They also less frequently had children, were more satisfied with their relationship and were less likely to plan a pregnancy within the next two years. Test-offer acceptors and decliners were comparable in age, religiosity, experiences with genetic counselling and testing, and having chronic and presumed hereditary conditions.

### Intention, barriers and views on this couple-based test-offer

Table 2 displays participants’ intentions, barriers to participation and views on couple-based test-offer.

**Table 2:** Intention, barriers and views on couple-based test-provision

Intention, barriers and views on couple-based test-provision	All *n* (%)	Test-offer acceptors *n* (%)	Test-offer decliners *n* (%)
Intention (survey 1)	*n* = 352 (6 missing)	*n* = 256 (3 missing)	*n* = 96 (3 missing)
Intention**			
Likely	306 (86.9)	240 (92.7)	66 (68.8)
Neutral	30 (10.6)	15 (5.8)	15 (15.6)
Unlikely	19 (5.4)	4 (1.5)	15 (15.6)
Intention (survey 2) (only participants who did not have ECS testing)	*n *= 54	*n *= 9	*n *= 45
Not having the test was a ‘deliberate’ decision			
Yes	33 (61.1)	8 (88.9)	25 (55.6)
No	21 (38.9)	1 (11.1)	20 (44.4)
If not, the reason for this was:			
We could not come to a common decision as a couple	0 (0)	0 (0)	0 (0)
It just did not happen	7 (33.3)	0 (0)	7 (35.0)
I had not thought about it anymore	2 (9.5)	0 (0)	2 (10.0)
It was not possible to be present at the GP appointment together	6 (28.6)	0 (0)	6 (30.0)
Other, such as pregnancy	6 (28.6)	1 (100)	5 (25.0)
Barriers (survey 1)	*n* = 348 (10 missing)	*n* = 256 (3 missing)	*n* = 92 (7 missing)
I think that test-participation takes a lot of time**			
Totally disagree	69 (19.8)	53 (20.7)	16 (17.4)
Disagree	149 (42.8)	123 (48.0)	26 (28.3)
Agree nor disagree	99 (28.4)	65 (25.4)	34 (37.0)
Agree	28 (8.0)	15 (5.9)	13 (14.1)
Totally agree	3 (0.9%)	0 (0)	3 (3.3)
I think that test-participation takes a lot of effort**			
Totally disagree	71 (20.4)	57 (22.3)	14 (15.2)
Disagree	174 (50.0)	135 (52.7)	39 (42.4)
Agree nor disagree	83 (23.9)	55 (21.5)	28 (30.4)
Agree	17 (4.9)	8 (3.1)	9 (9.8)
Totally agree	3 (0.9)	1 (0.4)	2 (2.2)
I think having to make a GP appointment before test-participation is a barrier**			
Totally disagree	55 (15.8)	48 (34.4)	7 (7.6)
Disagree	126 (36.2)	98 (38.3)	28 (30.4)
Agree nor disagree	85 (24.4)	60 (23.4)	25 (27.2)
Agree	65 (18.7)	40 (15.6)	25 (27.2)
Totally agree	17 (4.9)	10 (3.9)	7 (7.6)
I think having to give a blood sample is a barrier			
Totally disagree	113 (32.5)	88 (34.4)	25 (27.2)
Disagree	129 (37.0)	93 (36.3)	36 (39.1)
Agree nor disagree	62 (17.8)	45 (17.6)	17 (18.5)
Agree	33 (9.5)	22 (8.6)	11 (12.0)
Totally agree	11 (3.2)	8 (3.1)	3 (3.3)
Views on couple-based test-provision (T2)	*n* = 221 (6 missing)	*n* = 177 (5 missing)	*n* = 44 (1 missing)
Preferences for disclosure of ECS results			
Couple results only	122 (53.7)	101 (57.1)	21 (47.7)
Individual results	32 (14.1)	27 (15.3)	5 (11.4)
No preference	52 (22.9)	40 (22.6)	12 (27.3)
Not sure	15 (6.6)	9 (5.1)	6 (13.6)

Test-offer acceptors and decliners were, where relevant, compared using *T*-tests for continuous variables and *χ*^2^ tests for categorical variables. A *p* value of 0.05 was considered statistically significant

**p value < 0.01

#### Intention

The majority (87%) of study participants had a positive intention towards test-participation, but test-offer acceptors rated their intention more often as ‘likely’ compared with test-offer decliners (93% vs. 69%). Forty-four percent of test-offer decliners indicated that the decision to decline the test-offer had not been a considered one, and the reasons they most often indicated to explain why they did not attend pre-test counselling were ‘it just had not happened’ (*n* = 7) or ‘it was not possible to make a GP appointment together with my partner’ (*n* = 6).

#### Barriers

Test-offer decliners indicated significantly more frequently that test-participation took a lot of time and effort. In addition, 20% of test-offer acceptors and 35% of test-offer decliners agreed or totally agreed with the statement that having to make a GP appointment was a barrier to their participation.

#### Views on couple-based test-provision

Fifty-seven percent of test-offer acceptors and forty-eight percent of test-offer decliners indicated that, if they had to indicate a single preference between couple results or individual results they would prefer to receive couple results. Fifteen percent of test-offer acceptors and eleven percent of decliners would prefer a test that would give them individual carrier states. Twenty-four percent of test-offer acceptors and twenty-seven percent of decliners had no preference and five percent of test-offer acceptors and fourteen percent of decliners were not sure what they preferred.

### Arguments for and against accepting the couple-based test-offer

Table 3 shows that sparing a child a life with a severe genetic condition was considered the single most important argument to (potentially) accept this ECS test (29.6%). Other arguments that participants chose as most important were that they felt they had a responsibility as future parents to have this test (18%) and that a good result would be a great relief (13.0%). The distribution of these arguments was about the same for test-offer acceptors and decliners. Examples of ‘other’ arguments participants provided in favour of accepting ECS were curiosity, for the benefit of science, and due to experiences with genetic conditions in the family. Table 4 shows that for the participating couples the most important argument against having this ECS test was that the test-result would not influence their decision to have children (26.5%). Again, the distribution between test-offer acceptors and test-offer decliners was similar (25% and 30%, respectively). Twenty-seven percent of test-offer acceptors and eighteen percent of test-offer decliners provided additional explanations as to why they would not want to have the ECS test, such as a worry that after a positive test-result they would decide not to have children at all. Some stated they did not see any reason why not to undergo couple-based ECS.

**Table 3:** The most important arguments in favour or having a couple-based ECS test

Arguments in favour of couple-based ECS	All *n* = 355 (3 missing)	Test-offer acceptors *n* = 259	Test-offer decliners *n* = 96 (3 missing)
I think that my partner and I as (future) parents have a responsibility to do this test	63 (17.7)	50 (19.3)	13 (13.5)
I want to spare our child a life with a severe hereditary disease	105 (29.6)	78 (30.1)	27 (28.1)
If the test shows that we together are not carriers, this would be a great relief	46 (13.0)	33 (12.7)	13 (13.5)
I want to prevent my partner and I having to take care of a child with a severe hereditary disease	38 (10.7)	33 (12.7)	5 (5.2)
I want to know in good time if our child is at risk so as not to be confronted by having to make a choice about a late abortion	38 (10.7)	28 (10.8)	10 (10.4)
I want to be able to prepare myself for having a child with a severe hereditary disease	36 (10.1)	23 (8.9)	13 (13.5)
I think that abortion should be prevented if possible	6 (1.7)	1 (0.4)	5 (5.2)
Other (e.g. to benefit science, previous experiences with genetic conditions in the family)	23 (6.5)	13 (5.0)	10 (10.4)

**Table 4:** The most important arguments against having a couple-based ECS test

Arguments against couple-based ECS	All *n* = 355 (3 missing)	Test-offer acceptors *n* = 259	Test-offer decliners *n* = 96 (3 missing)
I do not want to know if my partner and I are carriers	27 (7.6)	14 (5.4)	13 (13.5)
I am against selecting children by screening (such as in this test)	13 (3.7)	7 (2.7)	6 (6.3)
I am afraid that if we turn out to be carriers this will have consequences for my relationship	33 (9.3)	22 (8.5)	11 (11.5)
I am afraid that if we turn out to be carriers this will have consequences for my insurance policies	12 (3.4)	12 (4.6)	0 (0)
I am afraid that if we turn out to be carriers we will be regarded as people with a disease	7 (2.0)	7 (2.7)	0 (0)
I am afraid that if we turn out to be carriers this will be registered with the authorities	11 (2.8)	10 (3.9)	1 (1.0)
I am afraid that if we turn out to be carriers we will end up in a medical treadmill	46 (13.0)	35 (13.5)	11 (11.5)
The test-result will have no influence on my having children with my partner	94 (26.5)	65 (25.1)	29 (30.2)
A test would take away the romance of a pregnancy	19 (5.4)	12 (4.6)	7 (7.3)
By taking a test, becoming pregnant is no longer natural	6 (1.7)	5 (1.9)	1 (1.0)
Other contra arguments (e.g. I do not see any reason why not to accept the test-offer)	87 (24.5)	70 (27.0)	17 (17.7)

### Response cards

As Fig. 2 shows, 70 women who were eligible for study participation explained on the response card why they were not interested in taking part. The majority cited reasons against having couple-based ECS, rather than issues regarding declining study participation, such as a perception that ECS results in over-medicalization of pregnancy, health-related issues, no perceived need to be tested (yet) and anticipating anxiety about the impact of a positive test-result.

## Discussion

This paper presents the initial interest from women 18–40 years and their partners of the first offer of cost-free couple-based ECS by trained (Dutch) GPs to couples from the general population and identifies their characteristics, views and barriers in terms of access and acceptance. Our results demonstrate that ~3% of all women approached and 15% of the estimated target population attended pre-test counselling with their GP, that is, were test-offer acceptors, of whom 90% proceeded with the test.

A few other studies have looked at uptake for single/few genes carrier testing in the Dutch general population. Henneman et al. [10] reported a 25% rate of test-offer acceptance of GP-provided cystic fibrosis (CF) carrier screening and an acceptance rate of 10% when couples attended educational sessions. Lakeman et al. reported a test-offer acceptance rate of 3% in their study on ancestry-based hemoglobinopathies and CF carrier testing by GPs in the Netherlands [11]. Although uptake rates in our study are similar to those from Henneman et al. [10], differences in study design prevent direct comparison of results. Furthermore, Gilmore et al. reported a 66% decline rate amongst eligible women in genomic carrier screening for reproductive purposes after being asked by telephone to participate [15]. This suggests that more eligible women decided to participate than in our study. However, differences in study design, such as the mode of invitation, eligibility criteria of having had carrier testing previously, and the option to receive medically actionable secondary findings, preclude direct comparison. Uptake figures are informative because they demonstrate whether actual uptake reflects couples’ intentions and could highlight potential barriers in test accessibility. Although, as we stated above, our main purpose of offering ECS in a reproductive setting is to inform couples’ reproductive decisions [3, 4] and maximising uptake rates is not an aim in itself.

In line with the existing literature [11, 16], test-offer acceptance in our study was lower than stated intentions. Our results demonstrate that practical barriers likely played a role for some test-offer decliners in our study, in particular having to make an appointment with the GP together, which was partly due to the design of our study. It is preferable that any future nationwide large-scale test-offer should still include pre-test counselling, because couples prefer to discuss this type of testing with a health professional, their GP in particular, and because this has shown to be feasible and resulted in informed decisions [7]. Gilmore et al. also found that logistical barriers such as lack of time were reasons mentioned frequently for women to decline participation in genomic carrier screening [15]. In addition, Gilmore et al. suggest that healthy individuals might not feel as much need to overcome barriers to test-participation in comparison to affected populations (such as parents of children affected by a genetic condition), where test-participation is usually higher [15]. Opportunities to alleviate the impact of these barriers are available and include web consultations with GPs, consultations outside office hours, and targeted information materials or decision aids to increase efficiency of the pre-test counselling, as well as the possibility to consult genetics professionals when necessary.

Apart from practical barriers, there are alternative reasons why the uptake rates in our study may deviate from those expected in future nationwide large-scale implementation. Firstly, the study was conducted in the northern part of the Netherlands, an area where participation in reproductive/prenatal genetic testing is typically lower than other areas of the Netherlands [17]. In addition, design related issues other than attending GP counselling with both partners may have resulted in lower acceptance rates. Most notable examples are the study’s consent procedure where 29% of women interested in participation did not return the consent forms and the test-offer was conditional upon survey participation. Secondly, our test-offer was a new and one-time offer, as ECS is not (yet) part of routine preconception care. Given that not all pregnancies are planned and most couples access health services prenatally rather than prior to conception, offering ECS during pregnancy as well may improve access to care. Finally, given that our test was free of charge, we could not study to what extent co-payment might be a barrier to test-participation. Research indicates that whilst people are willing to pay for ECS [7, 18], the price people are willing to pay is often lower than the actual cost of the test itself; thus, financial barriers might also diminish access to care, particularly for couples with low income.

### Acceptors and decliners

The majority of study participants had a positive intention towards test-participation, and this was high even among test-decliners (69%). Test-offer acceptors and decliners in our study were comparable in terms of sociodemographic characteristics, health status and experiences with chronic or hereditary conditions. In contrast to Gilmore et al. and to our expectations, we did not find that participants having experiences with any kind of, self-defined (presumed) hereditary conditions more frequently accepted testing [15]. In our study, couples who had experiences with (presumed) hereditary conditions, may have associated this less with their reproductive risk of having children with the specific severe AR conditions in this ECS test. The difference may also partly be due to the possibility to receive medically actionable secondary findings, as Kauffman et al., reporting on the same study, found that participants’ main motivation was to obtain this general health information. Most experiences with (presumed) hereditary conditions are likely to be adult-onset disease [19].

Test-offer acceptors more often had a higher educational level than test-offer decliners, which was also higher than the Dutch general population [20]. Other studies on reproductive genetic counselling and testing show similar findings [15, 17, 21]. Acceptors also differed from decliners regarding their reproductive/relationship profile in this study: they less often already had children, had a higher relationship satisfaction, and were less likely to plan their pregnancy in the very near future. These differences may suggest selection bias, but unequal representation is only problematic when access to the ECS test and information leading to informed choice are not equally available to all couples planning a pregnancy. Further research on the determinants of test-offer acceptance including these aspects, couples’ decision-making and couple dynamics, may help to identify relevant subgroups of patients to tailor information strategies and remove barriers to test-participation. The intention rate for ECS testing of participants in this study (87%) was more than double the intention rate in our previous survey study investigating couple-based ECS testing in a representative sample from the general population (34%) [7]. We therefore identified a subsection of this population who would like to make use of couple-based ECS when it was made available to them through the GP free of charge, but it is unlikely that the participants’ characteristics are generalisable to all couples of reproductive age.

### Reasons to accept and decline

We asked all participants to indicate their reasons for or against taking part in ECS, regardless of whether they chose to accept the test-offer. The reasons test-offer acceptors and test-offer decliners considered most important for or against ECS did not differ much. This was unexpected, but an explanation for these similarities might be the relatively homogenous study sample of which most started with the intention to accept this couple-based ECS test-offer. At the same time, we were also interested in understanding why not all eligible couples were interested in taking part. The explanations on the response cards reflected a variety of ethical, personal and practical arguments. Given that we only had access to a small group of those eligible women who decided not to take part in the study, more in depth exploration of motivations for undertaking and not undertaking ECS in the general population could be helpful to gain a better understanding of the desirability of offering ECS and potential barriers preventing all eligible couples from accessing ECS. The most important reason for participants to accept (to spare a child a life with a severe genetic condition) or decline testing (the test would not alter their reproductive plans) are in line with the literature [3, 7, 22] and align with the current aim of offering ECS testing (enhancing couples’ reproductive choice); participants’ reasons to decline the test-offer were not based on misunderstanding about the purpose of the test, or fears of discrimination or stigmatisation. As second most important reason to accept couple-based ECS, 18% of participants considered this their responsibility as a future parent. The perceived feeling of responsibility as a future parent to undergo ECS, is also brought forward in the paper of Van der Hout et al., who discuss this should be included as an aim for a (preconception) ECS test-offer alongside reproductive choice [23].

If they have to indicate a single preference after being informed about the aims of ECS, the majority of study participants indicated a preference for a couple-based approach over disclosing individual results. These results underline the findings of our previous study amongst potential users about couples’ views on couple-based ECS [6]. It should be noted that the response rate of survey 2 was relatively low in comparison to that of survey 1. Most of the drop-outs were test-offer decliners, who had a 45% response rate compared with 70% for test-offer acceptors. This means that the findings from survey 2 should be viewed with caution regarding the views of test-offer decliners. Regarding potential differences, participants with a higher educational level were more likely to respond to survey 2.

Given that it is the combined ‘couple-result’ which conveys information for reproductive decision-making, arguably, ECS couple-testing would be the new approach to offer carrier screening for AR conditions to the general population. This couple-based ECS test-offer could be complemented with individual carrier screening for X-linked conditions in the future. In this study, we focused on couple-based ECS as a free of charge test-offer in the Dutch public health system. We acknowledge that currently, ECS is not yet equally available and/or affordable to all couples planning a pregnancy. That is why, in certain contexts, arguments for couple-based testing or reporting individual carrier states may be different, such as for high frequency conditions in certain populations especially when cascade testing is reimbursed and population-based ECS is not (yet), when using whole exome sequencing in consanguineous populations and for ECS in a private setting.

## Conclusion

This study demonstrated that at least 15% of previously uninformed couples planning a pregnancy albeit a selective part, were interested and accepted the offer of a free, GP-provided couple-based ECS test. Lowering practical barriers, as identified in this study, leading to a test-offer that is easily and equally available to all couples planning a pregnancy could facilitate access for those with the intention to participate. Understanding the determinants for test-uptake and the barriers for non-participation of interested couples are necessary for the development of health policy and can inform future implementation of ECS in different settings.
